# Particulate matters from diesel heavy duty trucks exhaust versus cigarettes emissions: a new educational antismoking instrument

**DOI:** 10.1186/s40248-016-0042-7

**Published:** 2016-01-22

**Authors:** Cinzia De Marco, Ario Alberto Ruprecht, Paolo Pozzi, Elena Munarini, Anna Chiara Ogliari, Roberto Mazza, Roberto Boffi

**Affiliations:** 1 Tobacco Control Unit, Fondazione IRCCS Istituto Nazionale dei Tumori, Milan, Italy; 2 Patient Information Service, Fondazione IRCCS Istituto Nazionale dei Tumori, Milan, Italy

**Keywords:** Educational perspective, Smoking cessation, Second hand smoke

## Abstract

**Background:**

Indoor smoking in public places and workplaces is forbidden in Italy since 2003, but some health concerns are arising from outdoor secondhand smoke (SHS) exposure for non-smokers. One of the biggest Italian Steel Manufacturer, with several factories in Italy and abroad, the Marcegaglia Group, recently introduced the outdoor smoking ban within the perimeter of all their factories. In order to encourage their smoker employees to quit, the Marcegaglia management decided to set up an educational framework by measuring the PM_1_, PM_2.5_ and PM_10_ emissions from heavy duty trucks and to compare them with the emissions of cigarettes in an indoor controlled environment under the same conditions.

**Methods:**

The exhaust pipe of two trucks powered by a diesel engine of about 13.000/14.000 cc^3^ were connected with a flexible hose to a hole in the window of a container of 36 m^3^ volume used as field office. The trucks operated idling for 8 min and then, after adequate office ventilation, a smoker smoked a cigarette. Particulate matter emission was thereafter analyzed.

**Results:**

Cigarette pollution was much higher than the heavy duty truck one. Mean of the two tests was: PM_1_ truck 125.0(47.0), cigarettes 231.7(90.9) *p* = 0.002; PM_2.5_ truck 250.8(98.7), cigarettes 591.8(306.1) *p* = 0.006; PM_10_ truck 255.8(52.4), cigarettes 624.0(321.6) *p* = 0.002.

**Conclusions:**

Our findings may be important for policies that aim reducing outdoor SHS exposure. They may also help smokers to quit tobacco dependence by giving them an educational perspective that rebuts the common alibi that traffic pollution is more dangerous than cigarettes pollution.

## Background

Since January 2003 the Italian Government declared a national ban for cigarette smoking in indoor public places and workplaces, but nowadays some health concerns are arising from outdoor secondhand smoke (SHS) exposure for non-smokers, particularly near the public place entrances such as restaurants, bars, facilities of big industrial factories and hospital venues. Several studies in literature confirm the contribution of SHS to the outdoor environmental pollution and to poor air quality [1–11].

Health concerns regard not only never smokers, but also smoker employees. Consequently, some companies are considering a smoking ban that encloses the outdoor places within their facilities, in order to boost smoking cessation attempts among their smokers, thus improving their health conditions.

One of the biggest Italian Steel Manufacturer, with several factories in Italy and abroad, the Marcegaglia Group, within the frame of a wider project finalized to improve the safety of its workers, recently introduced the outdoor smoking ban within the perimeter of their premise in Ravenna. The data selected for ban implementation was March 21st, 2014, to highlight and transmit the message of the importance of breathing pure air in the first day of spring.

However, in order to strengthen the belief of hundreds smokers about the benefits of quitting smoking and to confer an educational perspective to their initiative, the Marcegaglia management asked for the support and trusted the experience of the Tobacco Control Unit (TCU) of the IRCCS Istituto Nazionale dei Tumori in Milan.

An educational experiment was set up.

## Methods

The first step of the TCU was to administer a questionnaire.

The questionnaire took into account gender, age, smoking habit (smoking status, number of cigarette smoked, smoking cessation attempts), attitudes toward smoking cessation activities and smoking ban proposed by the firm of all workers.

The second step was to set up an educational experiment by measuring the PM_1_, PM_2.5_ and PM_10_ emissions from a diesel heavy duty truck and to compare them with the emissions of a cigarette in an indoor controlled environment under the same conditions.

The essay was performed directly *in loco*, by the Marcegaglia Steel Factory in Ravenna. This factory is the most important center for the production and the distribution of steel products of the Marcegaglia Group and is extended over 550,000 square meters and employs over 700 workers.

The exhaust pipe of two trucks powered by a diesel engine of about 13,000/14,000cc^3^ were connected with a flexible hose to a hole in the window of a container of 36 m^3^ volume used as a field office. With door and window closed, the air exchange per hours (ACH) was calculated to be about 0.5/0.7.

Inside the office a fan was installed to assure the highest mixing factor as possible and one gravimetrically pre-calibrated Optical Particle Counter in mass (model Aerocet 531, Metone Instruments Inc.) was used to measure PM_1_, PM_2.5_ and PM_10_. The gravimetric calibration of the Aerocet 531 was performed by comparison with a Beta Attenuation Monitor model BAM-1020 of Metone Instruments Inc. with US EPA EQPM-0798-122 and German T.Ü.V. 936/21205333/A certification for PM_2.5_ and PM_10_ [12]. Gravimetric calibration of PM_1_ was not performed and the factory default gravimetric factor was used.

The heavy duty truck engines were supplied with common low sulphur diesel fuel oil purchased normally by the drivers on gas stations.

Both trucks were equipped with exhaust filters. Truck 1 had mileage of more than 1,000,000km but had recently a complete engine revision, while the Truck 2 was almost new. Before each measurement, the truck was started and run for a while far and downwind from the field office to allow the engines to warm up in order to avoid background PM level increase inside the office because of the cold start.

In the meantime the background level of PM inside the office was measured. Then, when the engine was sufficiently warm, the truck approached the office, the engine stopped for a few minutes to allow the quick connection of the tailpipe with the flexible hose to the window inlet, the door was closed and the diesel engine was started again, run idle for 8 min and then stopped. The door was opened and the office ventilated until the background level was reached. Then a volunteer current smoker, normally working and smoking inside the office, entered and smoked two cigarettes *at libitum* for 8 min with the door closed. The cigarette brand was Marlboro.

At the end of the 8 min the door was opened and again the office was ventilated until the background level was reached again. The background outdoor level remained relatively constant during the whole duration of the tests with small concentration changes with statistically not significant difference. Both tests were repeated two times.

## Results

The results of the questionnaire showed a worrying smoking prevalence of 43 % among all workers. This value is very significant if compared to the average smoking prevalence of the Italian population, close to 20 % [13]. Fifty-six percent of the current smokers were smoking more than 20 cigarettes/day and the working place was the place where they habitually smoked more than half of the cigarettes. Despite this high prevalence of current smokers, the vast majority (>80 %) of the workers appreciated the initiative and 42 % of the smokers declared that they would like to quit.

A minority of employees, mainly smokers, criticized the outdoor smoking ban. They declared that an outdoor smoking prohibition in areas where heavy trucks continuously load and unload steel products wouldn’t have reduced significantly the environmental air pollution and consequently their exposure to health hazards.

The results of the educational essay where therefore notified to all workers.

For the diesel engine/cigarettes emissions comparison two parameters were considered: a) the mean value of the background PM concentrations calculated about 8 to 10 min before starting the truck engines and/or to start to smoke the cigarettes and after the engine stop and/or stop to smoke for about 8 to 10 min after the complete washout of the office. b) the mean value of the concentrations calculated from the moment of the engines and/or cigarettes start until the maximum peak reached before the stop.

**Fig. 1:**
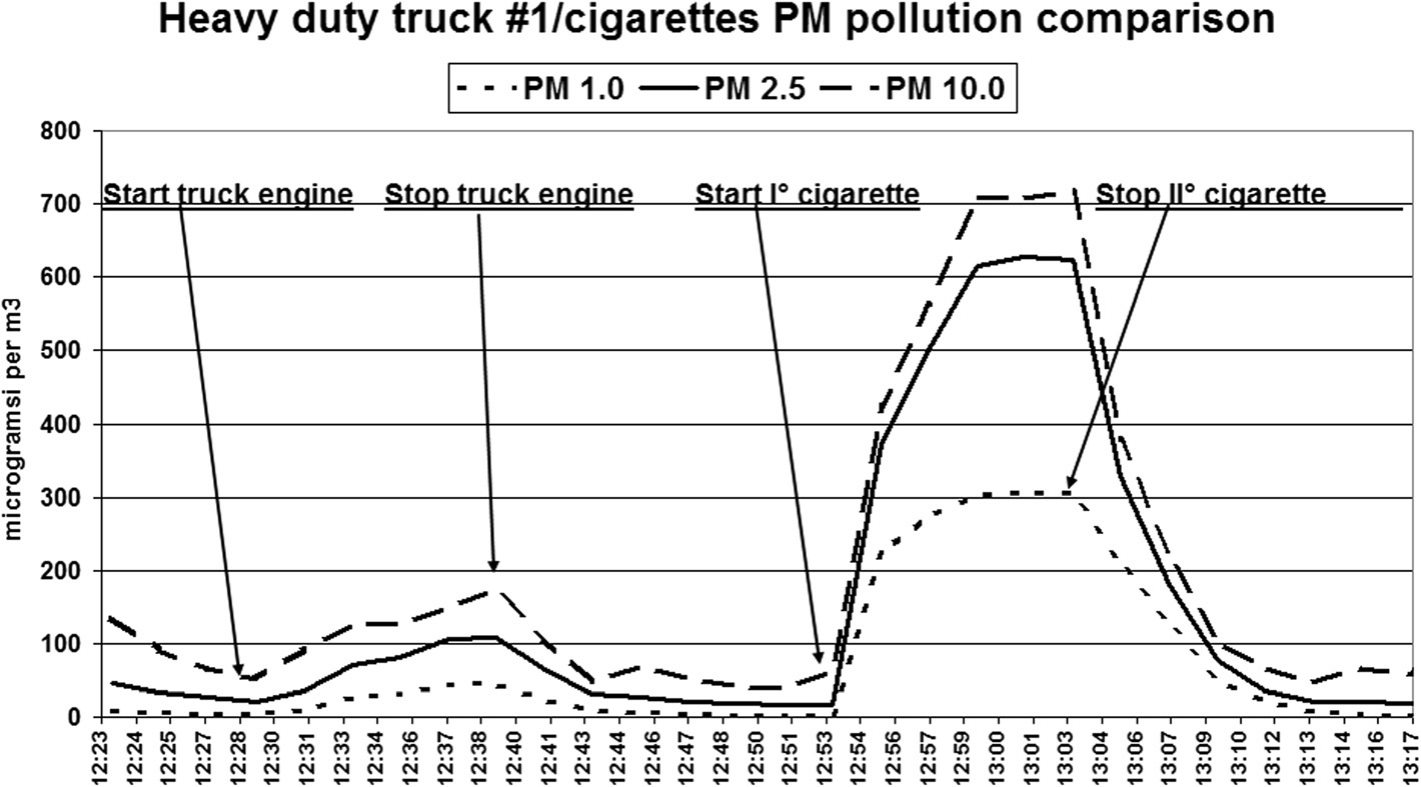
Real time graph of the PM1, PM2.5 and PM10 records of test truck 1
- Truck # 2: PM_1_ truck 218.8(86.9), cigarettes 297.0(15.2) *p* = 0.010; PM_2.5_ truck 441.5(190.0), cigarettes 1004.6(207.7) *p* = 0.006; PM_10_ truck 476.7(177.7), cigarettes 1029.5(207.7) *p* = 0.006 (see Fig. 2).

The tests showed the following values in μg/m^3^ (SD) background subtracted:

- Truck # 1: PM_1_ truck 32.3(9.4), cigarettes 271.2(38.6) *p* = 0.003; PM_2.5_ truck 65.0(19.1), cigarettes 507.6(119.2) *p* = 0.007; PM_10_ truck 69.2(22.7), cigarettes 547.2(136.2) *p* = 0.011 (see Fig. 1).

**Fig. 2:**
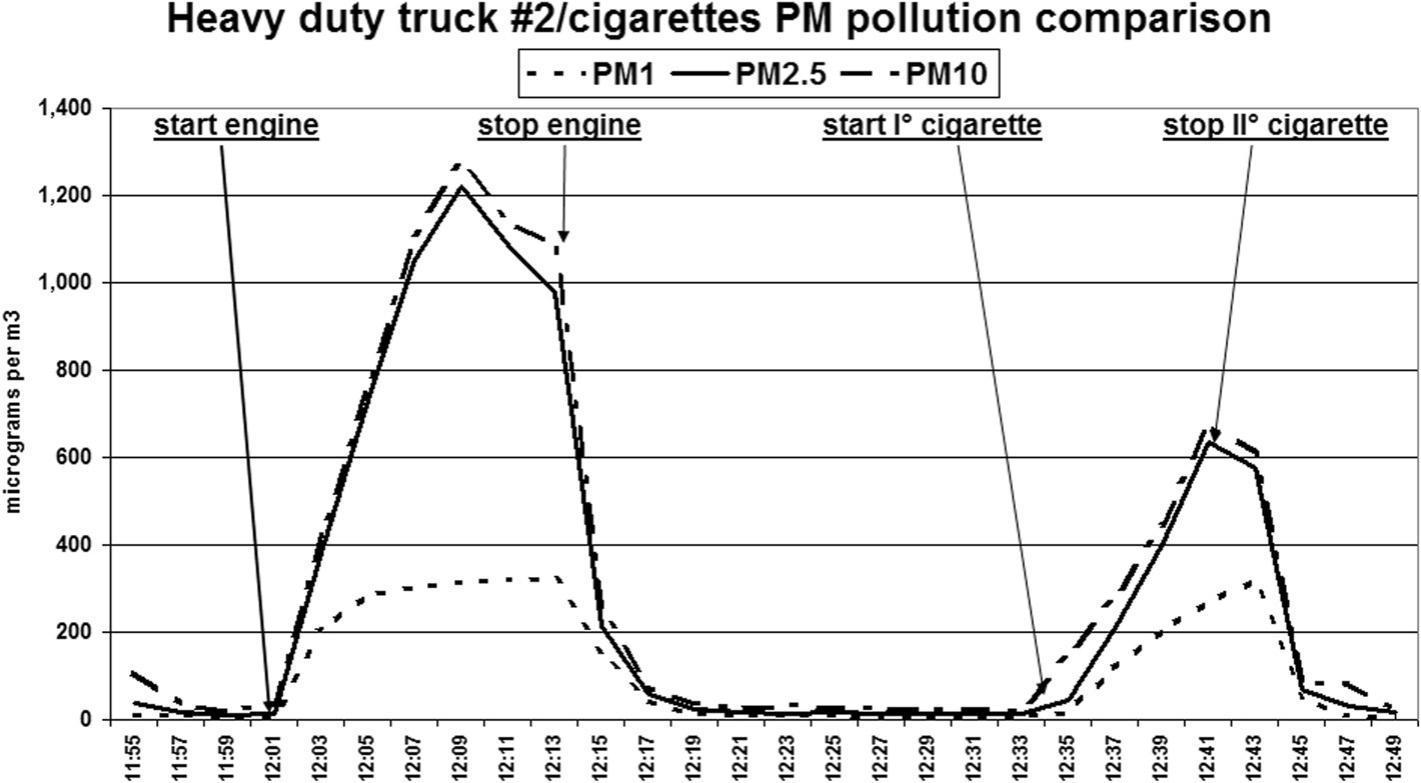
Real time graph of the PM1, PM2.5 and PM10 records of test truck 2
- Mean of the two tests: PM_1_ truck 125.0(47.0), cigarettes 231.7(90.9) *p* = 0.002; PM_2.5_ truck 250.8(98.7), cigarettes 591.8(306.1) *p* = 0.006; PM_10_ truck 255.8(52.4), cigarettes 624.0(321.6) *p* = 0.002 (see Fig. 3).

**Fig. 3:**
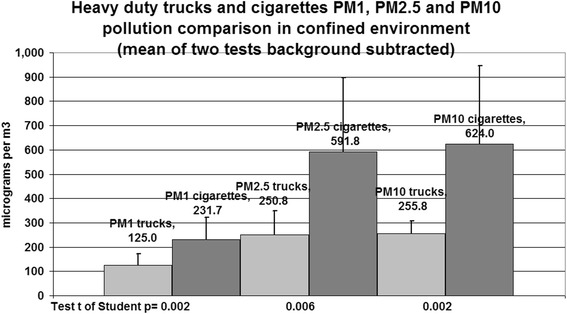
PM1, PM2.5 and PM10 means of the two tests background subtracted

## Discussion and conclusion

This study shows important data and bears educational messages.

First of all we were able to demonstrate that cigarette smoke generates more pollution in terms of PM_1_, PM_2.5_ and PM_10_ than a heavy duty truck. This finding was repeated in two tests and with two different generations of trucks, with a statistical significance (*p* < 0.05 between cigarettes and both trucks in all PM classes).

Our findings enforce the results of a recent study, where researchers depicted the exposure levels to fine and ultrafine particles (UFP) by a bus stop, in close proximity of a smoker. PM_2.5_ and UFP concentrations were 16–35 and 6.2 times higher than the background concentrations due to cars and trucks on an adjacent arterial highway [14].

We have already showed that environmental tobacco smoke is a major source of PM pollution, contributing to indoor PM concentrations up to 10-fold those emitted from an idling eco-diesel engine car [15].

Involuntary exposure to secondhand or environmental tobacco smoke has been declared as carcinogenic to humans (Group 1) according to the WHO-IARC Monograph [16].

Diesel engines exhaust has also been declared carcinogenic by the same agencies [17].

Our study shows for the first time that the amplitude of peak exposure to carcinogens may be unexpectedly higher for cigarette smoke, as compared to heavy trucks, even in outdoor and occupational settings such as the one of Marcegaglia Steel Factory.

A limitation of the study may be the limited number of experiments, due to the two heavy trucks availability in a single day, when the assay was performed. Nevertheless, we believe that the shocking message and its scientific valence do not look resized even if we were not able to perform multiple analysis.

An acute inflammatory response take place in the airways and peripheral blood of humans after short-term exposure to high levels of PM produced and a consistent increased risk for cardiovascular events occurs after both short and long-term exposure to PM air pollution by diesel exhaust [18, 19]. In the light of our findings, we find even more appropriate to protect healthy workers from the hazards of outdoor smoking.

It is notable that by using the US EPA Air Quality Index calculations (available at AQI http://airnow.gov/index.cfm?action=aqibasics.aqi), the PM2.5 maximum concentrations reached by the truck exhaust (441.5 μg/m^3^) would have been classified as hazardous [20].

On the other hand, the cigarette maximum concentrations (1004.6 μg/m^3^) would have been considered out of range by this calculation because higher than 500 μg/m^3^, therefore being extremely dangerous for health [21].

Our data have been used and are still used by the Marcegaglia Ravenna’s factory in health framework campaigns in order to strengthen the belief of hundreds smokers about the benefits of quitting smoking and to confer an educational perspective to their initiative.

Thanks to the Marcegaglia management a You Tube Video became available one year ago at https://www.youtube.com/watch?v=71qxTB0uOhI for everyone. An Editorial published on the “Eur J Paediatr Dent” has emphasized the educational importance of this study [22].

We believe that our findings may be important for policies that aim at reducing outdoor SHS exposure for sensitive categories (e.g., elderly, children, asthmatic subjects) and are intended to rebut the common alibi of smokers that traffic pollution is more dangerous than cigarettes smoking.

Initiatives such as the one here depicted may really boost the idea that quit smoking is worthless.
