# Erratum to: Particulate matters from diesel heavy duty trucks exhaust versus cigarettes emissions: a new educational antismoking instrument

**DOI:** 10.1186/s40248-016-0048-1

**Published:** 2016-02-16

**Authors:** Cinzia De Marco, Ario Alberto Ruprecht, Paolo Pozzi, Elena Munarini, Anna Chiara Ogliari, Roberto Mazza, Roberto Boffi

**Affiliations:** Tobacco Control Unit, Fondazione IRCCS Istituto Nazionale dei Tumori, Milan, Italy; Patient Information Service, Fondazione IRCCS Istituto Nazionale dei Tumori, Milan, Italy

After publication of the original article [1] it was brought to our attention that the graph in figure two (here referred to as Fig. 1) contained an error in the intertitles. The corrected graph has been included in this erratum as Fig. 1. Please note that this error does not affect any conclusions drawn in the original article.

**Fig. 1:**
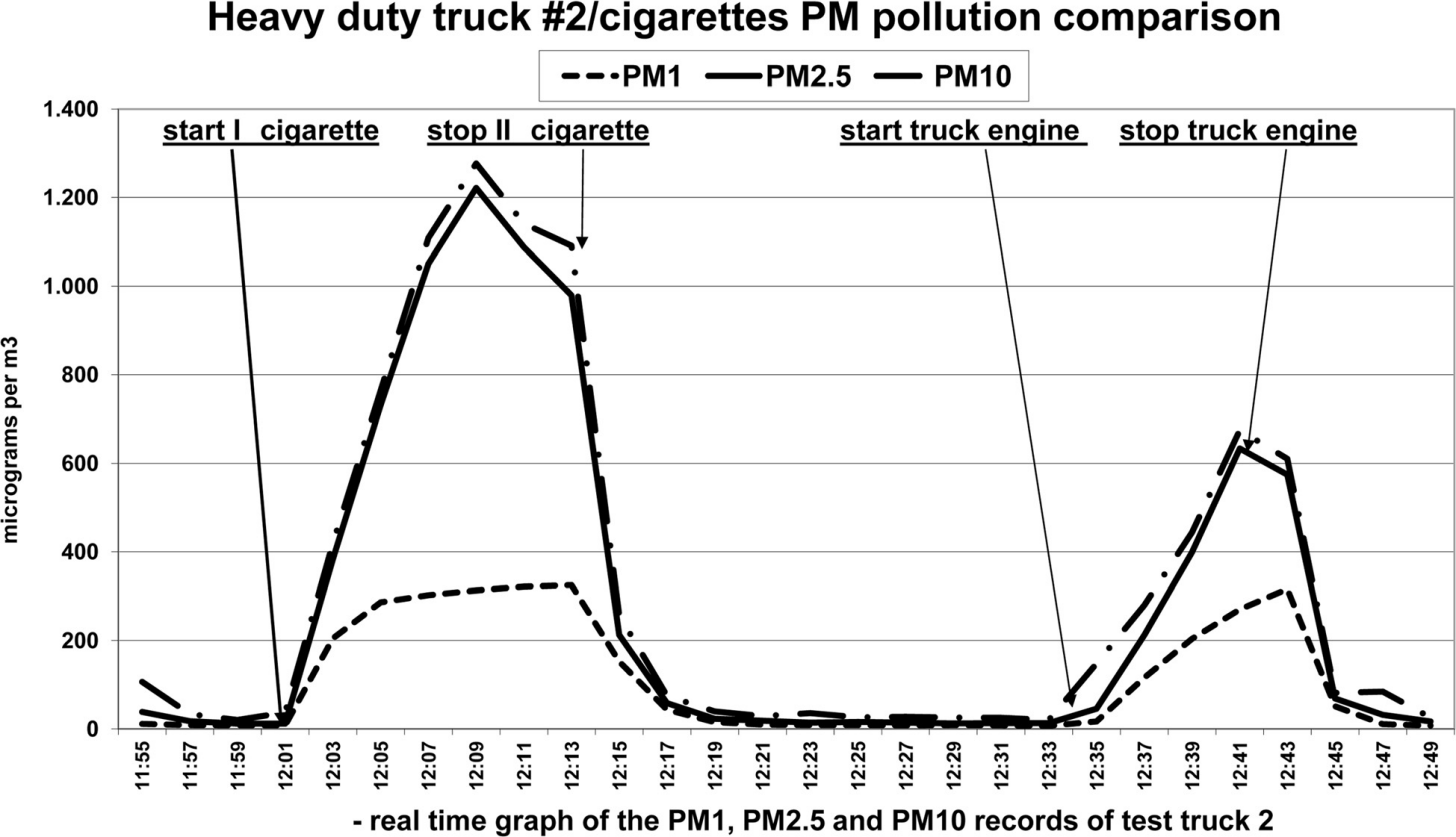
Real time graph of the PM1, PM2.5 and PM10 records of test truck 2
